# Clinical spectrum of females with *HCCS* mutation: from no clinical signs to a neonatal lethal form of the microphthalmia with linear skin defects (MLS) syndrome

**DOI:** 10.1186/1750-1172-9-53

**Published:** 2014-04-15

**Authors:** Vanessa A van Rahden, Isabella Rau, Sigrid Fuchs, Friederike K Kosyna, Hiram Larangeira de Almeida, Helen Fryssira, Bertrand Isidor, Anna Jauch, Madeleine Joubert, Augusta M A Lachmeijer, Christiane Zweier, Ute Moog, Kerstin Kutsche

**Affiliations:** 1 Institute of Human Genetics, University Medical Center Hamburg-Eppendorf, Martinistraße 52, 20246 Hamburg, Germany; 2 Current address: Department of Physiology, University of Lübeck, Lübeck, Germany; 3 Federal and Catholic University of Pelotas, Pelotas, Brazil; 4 Medical Genetics, School of Medicine, University of Athens, ‘Aghia Sophia’ Children’s Hospital, Goudi, Athens, Greece; 5 Unité de Génétique Clinique, CHU Nantes, Nantes, France; 6 INSERM, UMR-S 957, Nantes, France; 7 Institute of Human Genetics, Heidelberg University, Heidelberg, Germany; 8 Department of Pathology, CHU Nantes, Nantes, France; 9 Department of Clinical Genetics, VU University Medical Center, Amsterdam, The Netherlands; 10 Institute of Human Genetics, Friedrich-Alexander-Universität Erlangen-Nürnberg, Erlangen, Germany

**Keywords:** *HCCS*, Microphthalmia, X-linked, Linear skin defects, X chromosome inactivation

## Abstract

**Background:**

Segmental Xp22.2 monosomy or a heterozygous *HCCS* mutation is associated with the microphthalmia with linear skin defects (MLS) or MIDAS (microphthalmia, dermal aplasia, and sclerocornea) syndrome, an X-linked disorder with male lethality. *HCCS* encodes the holocytochrome *c*-type synthase involved in mitochondrial oxidative phosphorylation (OXPHOS) and programmed cell death.

**Methods:**

We characterized the X-chromosomal abnormality encompassing *HCCS* or an intragenic mutation in this gene in six new female patients with an MLS phenotype by cytogenetic analysis, fluorescence *in situ* hybridization, sequencing, and quantitative real-time PCR. The X chromosome inactivation (XCI) pattern was determined and clinical data of the patients were reviewed.

**Results:**

Two terminal Xp deletions of ≥11.2 Mb, two submicroscopic copy number losses, one of ~850 kb and one of ≥3 Mb, all covering *HCCS*, 1 nonsense, and one mosaic 2-bp deletion in *HCCS* are reported. All females had a completely (>98:2) or slightly skewed (82:18) XCI pattern. The most consistent clinical features were microphthalmia/anophthalmia and sclerocornea/corneal opacity in all patients and congenital linear skin defects in 4/6. Additional manifestations included various ocular anomalies, cardiac defects, brain imaging abnormalities, microcephaly, postnatal growth retardation, and facial dysmorphism. However, no obvious clinical sign was observed in three female carriers who were relatives of one patient.

**Conclusion:**

Our findings showed a wide phenotypic spectrum ranging from asymptomatic females with an *HCCS* mutation to patients with a neonatal lethal MLS form. Somatic mosaicism and the different ability of embryonic cells to cope with an OXPHOS defect and/or enhanced cell death upon *HCCS* deficiency likely underlie the great variability in phenotypes.

## Background

The microphthalmia with linear skin defects (MLS) syndrome (MIM 309801) is a rare X-linked neurodevelopmental disorder with male *in utero* lethality. The main clinical characteristics are uni- or bilateral microphthalmia and linear aplastic skin lesions which are usually limited to face and neck and develop into healed hyperpigmented areas with age. Additional features such as sclerocornea, corneal opacities, congenital heart defects, microcephaly, intellectual disability and agenesis of the corpus callosum have been observed less frequently [1].

The majority of MLS-affected patients carry a cytogenetically visible deletion or an unbalanced translocation leading to Xp22.2 monosomy [1]. After the minimal critical region of MLS syndrome had been defined to encompass the genes *MID1*, *HCCS* and *ARHGAP6* in 1994 [2,3], heterozygous intragenic mutations in *HCCS* were identified as causative in 2006 [4]. Since then, only one additional *HCCS* missense mutation has been described in a sporadic female patient with microphthalmia and sclerocornea of both eyes [5]. *HCCS* encodes the holocytochrome *c*-type synthase that is involved in mitochondrial oxidative phosphorylation (OXPHOS) where it catalyzes the incorporation of heme moieties to cytochrome *c* and cytochrome *c*_1_[6,7]. Cytochrome *c*_1_ is an integral component of complex III of the mitochondrial respiratory chain (MRC), while cytochrome *c* functions as an electron shuttle between complexes III and IV [8]. Complementation studies in yeast revealed severely impaired OXPHOS upon *HCCS* deficiency [6]. Recently, mutations in another gene, *COX7B* in chromosome band Xq21.1, encoding a structural subunit of cytochrome *c* oxidase (complex IV) involved in OXPHOS, have been identified in females with an MLS phenotype [9].

A high inter- and intrafamiliar phenotypic variability has been described in females with MLS syndrome [4,10-12]. Patients with an intragenic mutation or a (submicroscopic) deletion covering *HCCS* can show the full-blown MLS phenotype associated with other anomalies, the classical combination of microphthalmia/anophthalmia and linear skin defects, isolated ocular manifestations, aplastic skin areas restricted to face and neck with no additional abnormalities or no symptoms at all [4,5,10,11,13-15]. As possible explanations for this great clinical variability somatic mosaicism for the mutation and the degree of skewed X chromosome inactivation (XCI) in different tissues have been discussed [4,14,16,17].

Since 2007, we ascertained six novel female patients with a clinical diagnosis of MLS syndrome. Here we summarize the clinical and molecular data of these patients who were found to have different alterations involving the *HCCS* gene, ranging from classical chromosomal rearrangements of the Xp22 region to point mutations. We discuss different genetic mechanisms which protect females with an *HCCS* alteration from developing MLS-typical clinical features.

## Methods

### Patients

The study was approved by the Ethics Committee of the Medical Chamber of Hamburg (No. PV3585). We obtained clinical data as well as blood, buccal swabs, lymphoblastoid cells and/or DNA samples from six patients with a clinical diagnosis of MLS syndrome, who were assessed by experienced clinical geneticists. The clinical data and samples were obtained with informed consent, including consent to use the photographs in this report.

### Cytogenetic and fluorescence *in situ* hybridization (FISH) analysis

Conventional karyotyping was performed on metaphase spreads from peripheral blood lymphocytes by standard procedures. We used the Xp subtelomeric ToTelVysion probe (Abbott Molecular Inc, Des Plaines, IL, USA), the Vysis Steroid Sulfatase Deficiency probe (Vysis LSI STS; Abbott Molecular Inc) and the X centromere probe (Poseidon Satellite Enumeration Probe (SE) X (*DXZ1*), Kreatech Diagnostics, Amsterdam, NL) in FISH experiments. The bacterial artificial chromosome (BAC) clone RP11-163I1 (RPCI-11 human male BAC library) and fosmid clones (WIBR-2 human fosmid library [G248P8]) were received from the BACPAC Resource Center, Children’s Hospital Oakland, CA, USA. BAC and fosmid DNA was prepared using the NucleoBond Xtra Midi kit (Macherey-Nagel, Düren, Germany). BAC and fosmid DNA was labeled by nick translation using the CGH Nick Translation Kit and Spectrum Green-dUTP and Spectrum Red-dUTP (Vysis, Downers Grove, IL, USA), respectively, according to the protocol provided. Chromosomes were counterstained using 4′,6-diamidino-2-phenylindole (DAPI) (Serva Feinbiochemica, Heidelberg, Germany) and mounted in antifading solution (Vector Labs, Burlingame, CA, USA). Slides were analysed with a Leica Axioscope fluorescence microscope. Images were merged using a cooled CCD camera (Pieper, Schwerte, Germany) and CytoVision software (Applied Imaging, San Jose, CA, USA).

### Quantitative real-time polymerase chain reaction (qPCR)

qPCR of *HCCS* exons on genomic DNA was carried out as described previously [18]. Primer sequences are available on request.

### Sequencing of *HCCS*

DNA from whole blood, buccal swabs or lymphoblastoid cells was isolated by standard procedures. The coding region of the *HCCS* gene (exons 2–7; GenBank accession no. NM_005333.4) including flanking intronic sequences was amplified from genomic DNA. Primer sequences and PCR conditions are available on request. Amplicons were directly sequenced using the ABI BigDye Terminator Sequencing Kit (Applied Biosystems, Darmstadt, Germany) and an automated capillary sequencer (ABI 3500; Applied Biosystems). Sequence electropherograms were analysed using Sequence Pilot software (JSI Medical Systems, Kippenheim, Germany).

### Microsatellite analysis

Where mutations were shown to have arisen *de novo*, we verified declared relationships by genotyping both parents and the patient at fifteen short tandem repeat (STR) loci and *Amelogenin* using the The AmpFLSTR Identifiler PCR Amplification Kit (Applied Biosystems). DNA isolated from lymphocytes and the lymphoblastoid cell line of patient 3 was genotyped using the The AmpFLSTR Identifiler PCR Amplification Kit.

### Cloning of a mutation bearing PCR product to analyse for mosaicism

We cloned the amplicon of *HCCS* exon 6 in the pCR2.1 TOPO TA Cloning® Vector (Invitrogen, Karlsruhe, Germany). We picked bacterial clones and amplified them using standard protocols to identify those that contained a copy of the amplicon. We sequenced 138 PCR products to permit identification of those clones to contain mutation or wild-type bearing amplicons using the ABI BigDye Terminator Sequencing Kit (Applied Biosystems) and the automated capillary sequencer ABI 3500 (Applied Biosystems). Sequences were assembled and compared using the software SeqMan (DNASTAR, Madison, WI, USA).

### X chromosome inactivation analysis

The methylation status of the *AR, PGK1* or *MAOA* locus was examined by already described assays [19-21]. We modified the protocols as follows. For each DNA sample, two reactions were prepared. In the first reaction, 400 ng of DNA was digested with 8 U *Hpa*II in a total volume of 10 μl for 40 min at 37°C. In the second reaction, the same amount of DNA was incubated with the reaction buffer but without restriction enzyme. The digested and undigested fractions were submitted to PCR using fluorochrome-coupled primers (*AR*: forward primer: 5′-[6FAM]CTTTCCAGAATCTGTTCCAG-3′ and reverse primer: 5′-AAGGTTGCTGTTCCTCATC-3′; *PGK1*: forward primer: 5′-[6FAM]TGTTCCGCATTCTGCAAGCC-3′ and reverse primer: 5′-TATCCTTTTGTGCAGGAACC-3′; *MAOA*: forward primer: 5′-AGTAATCCTTTCCAGCTGCCGAC-3′ and reverse primer: 5′-[6FAM]TGCTTCATAAAGGGATTCTCTTTG-3′). PCR conditions are available on request. For *PGK1*: PCR products were digested with *Bst*XI at 55°C for 3 h and the enzyme was inactivated at 65°C for 20 min. For *MAOA*: PCR amplicons were precipitated with ethanol and 3 M NaAc (pH 5.8), and the precipitate was dissolved in H_2_O. After submitting the fraction to digestion with *Sac*I at 37°C for 1 h, the enzyme was inactivated at 65°C for 20 min. The resulting amplification products (*AR* locus) or digested fractions (*PGK1* and *MAOA* loci) were run on an ABI 3500 automated sequencer and the peak areas were calculated by GeneMapper Software v4.1 (Applied Biosystems). To account for preferential allele amplification, values for the digested DNA were normalized with those for the undigested DNA of each proband. The XCI pattern (expressed arbitrarily as a ratio of the smaller:larger allele) was calculated by applying the previously reported formula [22]: skewing = (d_1_/u_1_)/(d_1_/u_1_) + (d_2_/u_2_), where d_1_ and d_2_ represent the two raw peak area values of the digested sample, and u_1_ and u_2_ represent the raw peak area values of the undigested sample. In addition, one control male sample and one sample from a female known to have a completely skewed pattern of XCI (>98:2) were included in every batch of samples, to control for complete digestion and amplification efficiency.

X-inactivation testing in patient 3 was initiated at the respective centre within routine diagnostics.

## Results

In the six female patients with typical clinical manifestations of MLS syndrome, we identified heterozygous alterations of the *HCCS* gene. These comprised sequence-level mutations as well as microdeletions and cytogenetically visible deletions including multiple other genes (Table 1).

**Table 1:** Collection of molecular and clinical data of 6 patients with MLS syndrome

**Patients**	***HCCS *** **mutation**	**XCI**^**a**^	**Dermatologic findings**	**Ocular findings**			**Cardiac defects**	**CNS anomalies**	**Microcephaly**	**Developmental delay/intellectual disability**	**Facial dysmorphism**	**Short stature**	**Other anomalies**
				**Microphthalmia/anophthalmia**	**Sclerocornea/corneal opacity**	**Others**							
**1**	ish del(X)(p22.2p22.2), interstitial deletion of ~850 kb including *HCCS* inherited	100:0	―	bilateral microphthalmia	bilateral sclerocornea	gracile optic nerves and chiasma, bilateral microcornea, ectopic pupil (right), coloboma iris (right), anterior eye chamber defect	―	very mild delay in myelination	―	―	small deep-set eyes	―	―
**7 years Dutch**													
**2**	c.589C > T (p.R197*) *de novo*	98:2	linear skin defects on the neck	microphthalmia (Peter’s anomaly with adherence of iris on cornea and anterior chamber) (right), anophthalmia (left)	unilateral sclerocornea and corneal opacity (right)	optic nerve hypoplasia (left)	ventricular tachycardia, poor contraction of left ventricle, histiocytoid cardiomyopathy, eosinophilic cell infiltration	abnormal myelination, hypoplastic corpus callosum, absence of septum pellucidum	―	n.a.	―	-3 SD^b^	―
**Died at 4 months French**													
**3**	c.[=/524_525delAG] (p.[=/E175Vfs*30]) *de novo*	82:18	―	unilateral microphthalmia (left)	unilateral sclerocornea (left)	unilateral cornea plana (left)	―	n.d.	―	mild motor delay^c^	mild facial asymmetry, prominent philtrum	―	mild muscular hypotonia, sacral dimple
**3 years German**													
**4**	46,X,del(X)(p22) *de novo*	100:0	linear skin defects on the face, neck, hand (right) and foot (left)	severe bilateral microphthalmia	corneal opacity	aphakia	―	agenesis of corpus callosum	-4.86 SD^d^	+	mild prognathism	48 cm (0.1 cm <3rd centile)^e^; -3.33 SD^d^	―
**4 years German**													
**5**	46,X,del(X)(p22)* de novo*	100:0	linear skin defects on the face, small hemangiomas on face, neck and right hand	microphthalmia (left), anophthalmia (right)	unilateral sclerocornea (left)	―	―	―	49 cm (3rd centile)^f^	+	long thin face, mild prognathism, depressed nasal bridge, microdontia, high palate, low-set and posteriorly rotated ears	105 cm (3rd centile)^g^	deafness (right), anal atresia with ectopic anus and fistula, clinodactyly of the fifth finger
**10 years Greek**													
**6**	interstitial deletion of ≥3 Mb including *HCCS*^h^	100:0	linear skin defects on the face	bilateral microphthalmia	bilateral sclerocornea	―	―	―	―	―	―	67 cm^i^	intralesional absence of sebaceous glands (dermatoscopic examination)
**1 year Brazilian**													

**Legends:** n.a.: not applicable; n.d.: not determined; SD: standard deviation; ―: absent; +: present; ^a^: determined in leukocytes; ^b^: at the age of 3 months; ^c^: at the age of 11 months; ^d^: at the age of 3 years; ^e^: at birth; ^f^: at the age of 7 years; ^g^: at the age of 6 years; ^h^: inheritance of the interstitial microdeletion could not be determined as the father was not available. ^i^: at the age of 9 months.

### Chromosomal analysis and FISH

Routine cytogenetic analysis was performed in patients 1 and 3–6 diagnosed with MLS syndrome and revealed an apparently normal female karyotype in patients 1, 3, and 6. Patients 4 and 5 showed a structurally abnormal karyotype with 46,X,del(X)(p22) (Table 1). We confirmed the terminal Xp deletion by FISH using BAC RP11-163I1 encompassing the *HCCS* gene in patients 4 and 5 and a subtelomeric Xp probe together with a probe covering the *STS* gene (Xp22.32) in patient 4 (data not shown). The estimated minimum size of the two deletions was ≥11.2 Mb. FISH with the *STS* and X centromere probes on metaphase spreads of the parents of patient 4 revealed the expected number of signals (data not shown).

High resolution molecular karyotyping using Agilent Human Genome CGH 44 K oligonucleotide arrays (Agilent, Santa Clara, CA, USA) with the ISCA design (http://www.iscaconsortium.org) was performed on a clinical basis in patient 2 and revealed no disease-associated copy number variant (data not shown).

### Copy number analysis of *HCCS* by FISH and qPCR

In patient 5, the large Xp deletion was confirmed by qPCR of the selected *HCCS* exons 1, 3, 4, and 6 (Additional file 1: Figure S1). Expected values were observed for the relative copy number of the four *HCCS* exons in both parents of patient 5 (Additional file 1: Figure S1) indicating that her deletion occurred *de novo*.

**Figure 1:**
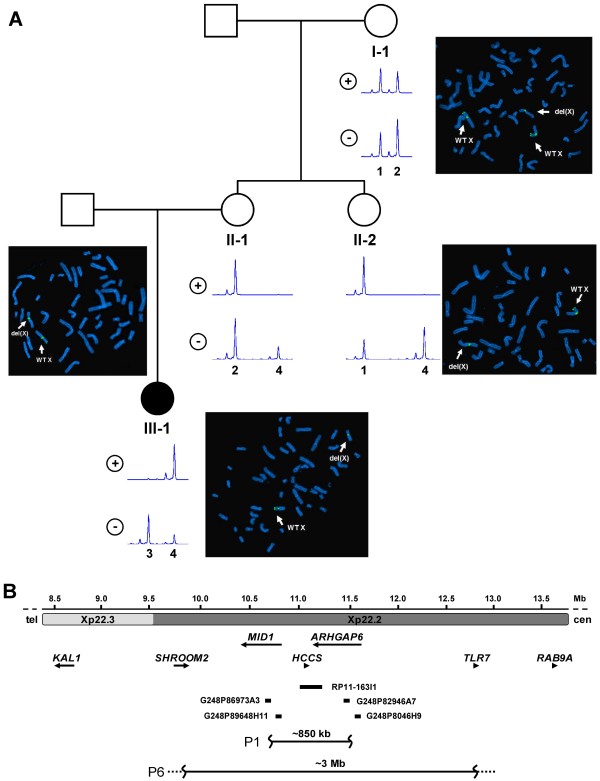
**Pedigree, FISH and X chromosome inactivation of patient 1 and three healthy female relatives. A**. Pedigree of patient’s 1 family. Patient 1 (III-1) is affected by MLS syndrome, while her mother (II-1), her maternal aunt (II-2) and her maternal grandmother (I-1) are asymptomatic. FISH analysis with BAC RP11-163I1, spanning the *HCCS* gene on metaphase spreads of the four females revealed one signal in patient 1 (III-1), her mother (II-1) and her aunt (II-2). For the maternal grandmother (I-1), two signals for RP11-163I1 and three signals for the X centromere probe *DXZ1* were obtained*.* BAC RP11-163I1 and *DXZ1* were labelled with Spectrum Green-dUTP. Arrows point to the wild-type X chromosome (WT X) and the X chromosome with the microdeletion at Xp22.2 (del(X)). X chromosome inactivation was determined by analysing the methylation status of the androgen receptor gene at Xq12. Predigestion of genomic DNA isolated from lymphocytes with and without *Hpa*II is indicated by (+) and (-), respectively. Representative electropherograms show the different *AR* alleles (designated as 1, 2, 3 and 4) in the four females. Females II-1, II-2 and III-1 have extremely skewed X inactivation (upper electropherograms indicated with +). **B**. Physical map of part of the Xp22.3 and Xp22.2 regions that are indicated by horizontal grey bars; Mbs and the telomere (tel) to centromere (cen) orientation are given. Arrows represent selected genes in Xp22 and gene symbols are given; arrowheads indicate the 5′ → 3′ transcription direction of the genes. BAC RP11-163I1 (RP11 Human BAC Library) and four Xp22.2 fosmid (WIBR-2 Human Fosmid Library) clones are indicated by black bars and names are given. Interstitial deletions found in patients 1 (P1) and 6 (P6) are depicted as horizontal black lines and the size of each deletion is given. Dotted and wavy lines indicate that the deletion breakpoints were not fine-mapped.

To uncover a possible microdeletion covering *HCCS*, we performed FISH with RP11-163I1 and detected only one signal in patient 1 (III-1 in the pedigree of Figure 1A) suggesting an interstitial Xp deletion. We delineated the breakpoints by FISH with Xp22.2 fosmid clones (Figure 1B). For the distal deletion breakpoint, two signals were obtained for G248P86973A3, while only one signal was detected for G248P89648H11 (Additional file 1: Figure S2). To map the proximal deletion breakpoint, we used fosmid G248P82946A7 which gave only one signal, while G248P8046H9 yielded two signals (Additional file 1: Figure S2). Thus, patient 1 carried a ~850 kb microdeletion covering the entire *HCCS* gene and part of the neighboring genes *MID1* and *ARHGAP6* (ish del(X)(p22.2p22.2)) (Figure 1B and Table 1). qPCR confirmed that the relative copy number of *HCCS* exons 2–7 in patient 1 was comparable to a haploid sample (Additional file 1: Figure S3). By FISH with BAC RP11-163I1 we detected the Xp22.2 microdeletion in the healthy mother (II-1 in Figure 1A) and the healthy maternal aunt of patient 1, too (II-2 in Figure 1A). In her healthy maternal grandmother, routine cytogenetic analysis revealed three X chromosomes in 25 analysed metaphases (data not shown). To confirm triple X syndrome, we performed FISH with RP11-163I1 in combination with the X centromere probe and detected two signals for the BAC and three for the centromere probe in 65 analysed metaphases (I-1 in Figure 1A and data not shown). However, in seven metaphases we found signals on two X chromosomes for the centromere probe and one signal for the BAC clone indicating mosaic trisomy X in patient’s 1 maternal grandmother (data not shown). By analysing six X-chromosomal microsatellite markers we confirmed the presence of three alleles in this female (data not shown). To summarize, patient’s 1 healthy maternal grandmother carried the ~850 kb microdeletion covering *HCCS* on one of her X chromosomes, and she had a mosaic form of triple X in her leukocytes (47,XXX.ish del(X)(p22.2p22.2)(HCCS-)[65]/46,XX.ish del(X)(p22.2p22.2)(HCCS-)[7]).

**Figure 2:**
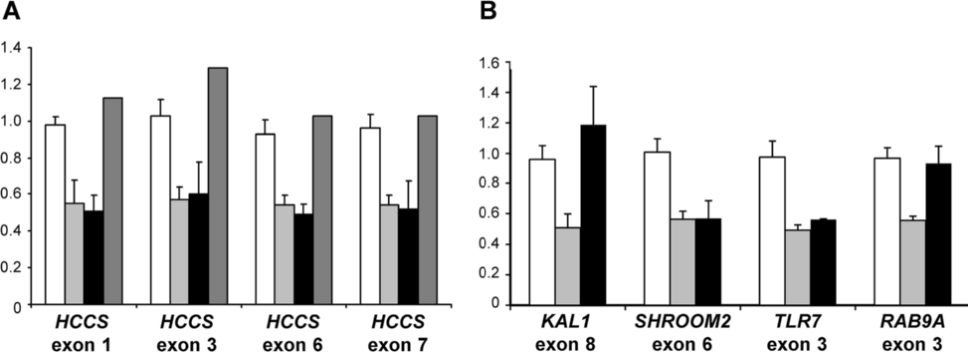
**Copy number analysis of *HCCS *and neighboring genes by quantitative real-time PCR in patient 6. A**. Relative quantification of copy number of *HCCS* exons 1, 3, 6 and 7 by qPCR on genomic DNA of patient 6 (black bars) and her mother (darkgrey bars) revealed values that are comparable to a haploid sample (lightgrey bars) and a diploid sample (white bars), respectively. White, lightgrey and black bars represent the mean ± SD of two independent experiments, each performed in duplicate. The darkgrey bars represent the mean of one experiment performed in duplicate for each exon. **B**. Relative quantification of copy number of *HCCS* surrounding genes in patient 6. qPCR for *KAL1* exon 8 and *RAB9A* exon 3 on genomic DNA of patient 6 (black bars) revealed values that were comparable to a diploid sample (white bars), while those for *SHROOM2* exon 6 and *TLR7* exon 3 (black bars) were comparable with a haploid sample (lightgrey bars). Each bar represents the mean ± SD of at least two experiments performed in duplicate.

To analyse for the presence of a possible (submicroscopic) Xp22 deletion in patient 6 from Brazil, we had to perform qPCR experiments as only DNA and no lymphocyte suspension culture was available. The relative copy number of the selected *HCCS* exons 1, 3, 6 and 7 was found to be comparable with a haploid sample in patient 6, while her mother yielded values comparable with a diploid sample (Figure 2A). A DNA sample of patient’s 6 father was not available. To estimate the approximate size of the deletion, we selected genes located telomeric and centromeric to *HCCS* and performed additional qPCRs. We mapped the proximal breakpoint between the two genes *TLR7* and *RAB9A* and the distal breakpoint between *KAL1* and *SHROOM2* (Figures 1B and 2B). The data demonstrates a submicroscopic interstitial Xp deletion of a minimum size of 3 Mb in patient 6 encompassing the entire genes *HCCS*, *MID1* and *ARHGAP6* (Figure 1B).

### Sequence analysis of *HCCS*

**Figure 3:**
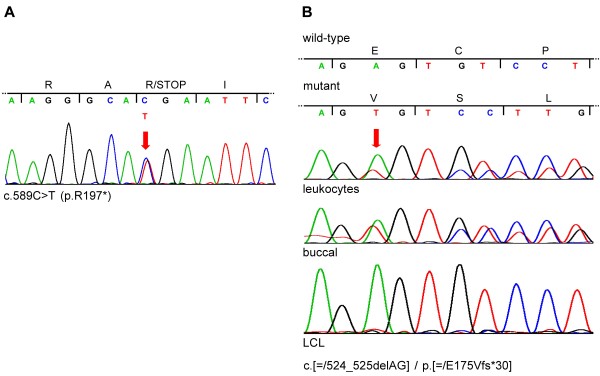
***HCCS *sequence-level mutations in patients 2 and 3. A**. Sequence electropherogram from genomic DNA of patient 2 showing part of *HCCS* exon 6. Nucleotide triplets and encoded amino acids (one letter code) are indicated above the electropherogram. The red arrow points to the double peak in the electropherogram showing heterozygosity for the nonsense mutation c.589C > T (p.R197*). **B**. Sequence electropherograms of part of *HCCS* exon 6 from DNA isolated from leukocytes (top), buccal cells (middle) and the lymphoblastoid cell line (LCL; bottom) of patient 3. Nucleotide triplets and encoded amino acids (one letter code) are shown for the wild-type and mutant allele above the electropherograms. The red arrow points to the first double peak in the electropherogram indicating the start of the frameshift. In LCL-derived DNA only *HCCS* wild-type sequence was visible in the electropherogram. Patient 3 carries the mosaic frameshift mutation c.[=/524_525delAG] / p.[=/E175Vfs*30] in *HCCS*.

We Sanger-sequenced the coding exons 2–7 of *HCCS* in patients 2 and 3. In patient 2, we identified the heterozygous *de novo* nonsense mutation c.589C > T (p.R197*) in exon 6 (Figure 3A) (paternity confirmed; data not shown). In leukocyte-derived DNA of patient 3, we detected the 2-bp deletion c.524_525delAG in exon 6 that results in a frameshift and introduction of a premature termination codon (p.E175Vfs*30) (Figure 3B). The sequence profile showed slightly lower signals for the mutant variant superimposed on the wild-type sequence suggesting that the mutation was present in the mosaic state (Figure 3B). To confirm somatic mosaicism of the 2-bp deletion in *HCCS* in patient 3, we amplified exon 6 from leukocyte-derived DNA, cloned the amplicon and subjected a total of 138 individual *E.coli* colonies to PCR followed by sequencing. We identified 102 wild-type alleles (73.9%) and 36 alleles with the mutation c.524_525delAG (26.1%) demonstrating somatic mosaicism in patient 3 (data not shown). Thus, patient 3 had a mixed population of *in vivo* lymphocytes: ~52% of lymphocytes carry the heterozygous c.524_525delAG mutation and ~48% carry two wild-type *HCCS* alleles. We next investigated DNA from two other cell types of patient 3 and sequenced *HCCS* exon 6 in DNA isolated from buccal cells and a lymphoblastoid cell line (LCL). As shown in Figure 3B, the peak height of the two sequence profiles representing the two different *HCCS* alleles in patient 3 was similar in buccal cell-derived DNA. In contrast, the sequencing pattern of the mutant *HCCS* allele is not any more visible in DNA isolated from LCLs (Figure 3B). Absence of the *HCCS* mutation in LCL-derived DNA suggested clonal evolution of the lymphoblastoid cell line of patient 3 [23,24]. By genotyping 16 genetic markers we confirmed that leukocyte- and LCL-derived DNA samples came from the same person (patient 3) (data not shown). Together, the data indicates that patient 3 carried a mosaic 2-bp deletion (c.[=/524_525delAG]/p.[=/E175Vfs*30]) in *HCCS*. Absence of the mutation in the parents is in line with somatic mosaicism in patient 3 (paternity confirmed; data not shown).

### XCI analysis

The XCI pattern was determined in all patients. A totally (100:0) and extremely skewed (98:2) XCI was detected at the *AR* locus in leukocyte-derived DNA of patients 1 and 6 and patient 2, respectively (Table 1). As the interstitial Xp22.2 deletion in patient 1 was also found in three healthy family members, we also determined their XCI pattern and found complete skewing (100:0) in leukocytes of the mother and maternal aunt at the *AR* locus (Figure 1A). In the maternal grandmother, we detected two X-chromosomal alleles prior and after restriction of the DNA with the methylation sensitive enzyme *Hpa*II (Figure 1A) suggesting that two of the three X chromosomes carry the same *AR* allele and cannot be distinguished. Thus, a skewed XCI ratio possibly escaped detection in this female. Patients 4 and 5 were not informative at the *AR* locus. In patient 4, total XCI skewing (100:0) was found at the *MAOA* locus (Table 1). Similarly, patient 5 had an XCI ratio of 100:0 at the *PGK* locus (Table 1). A slightly skewed XCI pattern (82:18) was identified in leukocyte-derived DNA of patient 3 at the *FMR1* locus that is in line with a mosaic *HCCS* mutation (Table 1).

### Clinical data

**Figure 4:**
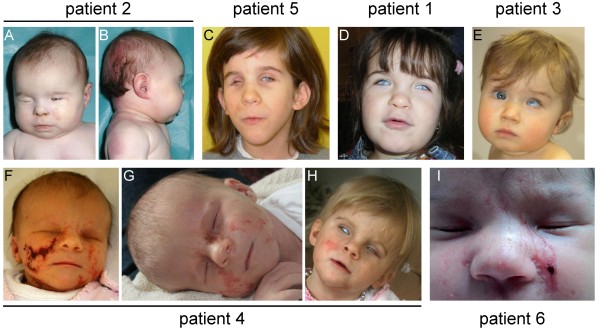
**Photographs of the six patients with MLS syndrome and a heterozygous *HCCS *mutation or Xp22 monosomy. A** and **B**. Patient 2 died at the age of 4 months. She presented with microphthalmia and sclerocornea of the right eye and anophthalmia of the left eye **(A)**. Linear skin defects were observed on her neck **(B). C**. In the 7-year-old patient 5, linear skin defects and small hemangiomas were noted. She showed microphthalmia and sclerocornea of the left eye and anophthalmia of the right eye. **D**. Microphthalmia and sclerocornea of both eyes were present in patient 1, while linear skin defects were absent. **E**. In patient 3 (age 11 months), microphthalmia and sclerocornea of the left eye were diagnosed. No linear skin defects were noted. **F**, **G** and **H**. Linear skin defects on the face of patient 4 were very prominent at birth (**F**, 4 days old), but healed with age (3 weeks old in **G** and 2 years old in **H**). Severe bilateral microphthalmia was observed in the patient. **I**. Bilateral microphthalmia and sclerocornea were observed in patient 6; she also had typical linear skin defects on her face (photographs submitted with written consent from the patients’ legal guardians for publication in print and online).

Clinical data of the six index patients were reviewed (Table 1). All were females aged between 4 months and 7 years at latest follow-up. Patient 2 died at the age of 4 months because of ventricular tachycardia and showed a histiocytoid cardiomyopathy on post-mortem pathological examination. The most consistent clinical features of all patients were unilateral/bilateral microphthalmia/anophthalmia and unilateral/bilateral sclerocornea/corneal opacity (Figure 4A, C-E, H and I). Additional ocular anomalies were frequently observed (Table 1). Four girls (patients 2 and 4–6) had linear skin defects at least on the face and neck (Figure 4A-C, F-I). Patient 4 presented with severe erythematous linear skin lesions on her face after birth (Figure 4F). They healed with age and developed to hyperpigmented areas (Figure 4G and H). Patients 1 and 3 did not show congenital skin lesions (Figure 4D and E). No cardiac defects except for those described in patient 2 were reported. Brain imaging studies revealed delayed/abnormal myelination in patients 1 and 2 and corpus callosum agenesis/hypoplasia in patients 2 and 4. Occipital frontal circumference in the low normal range or microcephaly was observed in patients 4 and 5. Individuals 2, 4, 5 and 6 had postnatal growth retardation of varying degree. Other anomalies (sacral dimple, deafness, anal atresia with ectopic anus and fistula, clinodactyly of fifth finger, and lack of sebaceous glands) were only seen in single patients. Minor craniofacial dysmorphism was observed in patients 1, 3, 4 and 5 (Table 1 and Figure 4C, D, E, and H).

## Discussion

We present molecular and clinical data of six new female patients with typical features of MLS syndrome and a deletion or mutation involving *HCCS*. MLS syndrome was first described more than 20 years ago as a disorder characterized by congenital linear skin lesions and microphthalmia [25,26]. Since then a total of 62 cases with this clinical diagnosis have been reported [1,27-31]. The majority of patients had a chromosomal abnormality resulting in monosomy for the Xp22.2 region. MLS syndrome mainly affects females, however, ten males with an XX karyotype and Y-chromosomal material or 46,XY and a mosaic inversion involving the band Xp22.2 are known [11,15,29,32-36]. Finally, *HCCS* turned out to be the culprit gene in Xp22.2 [4] and is implicated in all chromosomal rearrangements reported in MLS syndrome-affected individuals to date. The known intragenic *HCCS* mutations comprise an 8.6-kb copy number loss of part of the gene and the three point mutations c.589C > T (p.R197*), c.649C > T (p.R217C) and c.475G > A (p.E159K) [4,5]. By complementing a *Saccharomyces cerevisiae* strain deficient for the HCCS orthologue Cyc3p, the three sequence-level variants in *HCCS* were found to be loss-of-function mutations affecting MRC [4,5]. Additional evidence for implication of mitochondrial dysfunction in MLS syndrome came from the discovery of *COX7B* as the second gene for this neurocutaneous disorder; it encodes a structural subunit of MRC complex IV [9]. Deficiency of the COX7B or HCCS orthologue in medaka was found to recapitulate the MLS phenotype and demonstrated an essential function of the MRC complexes III and IV in human development in general and central nervous system (CNS) development in particular [9,37].

The *HCCS* mutations identified in the six patients reported here represent the full spectrum of genetic alterations leading to null alleles: two patients had the common terminal Xp deletion with a size of ≥11.2 Mb and two females carried a submicroscopic interstitial deletion, one of ~850 kb and the other of 3 Mb as minimum size. The four deletions contain multiple genes including *HCCS*. Only three cryptic interstitial deletions covering *HCCS* have been reported in MLS-affected females so far: One of >3 Mb [13], a second of 3.6 Mb [38] and a third of 185–220 kb [30]. The two remaining MLS-affected females carried *de novo* intragenic sequence changes, the nonsense mutation c.589C > T (p.R197*) which seems to represent a recurrent mutation [4], and the novel mosaic 2-bp deletion c.[=/524_525delAG] (p.[=/E175Vfs*30])]. Similar to other reports, we could not establish a genotype-phenotype correlation and observed a high intra- and interfamilial phenotypic variability in patients carrying an *HCCS* alteration [1].

Unilateral/bilateral microphthalmia/anophthalmia and sclerocornea/corneal opacity of one or both eyes were found in all patients (Table 1). Other ocular findings include a wide variety of anomalies, such as microcornea, coloboma, anterior chamber defect, optic nerve hypoplasia (this report) as well as retinal abnormalities, congenital glaucoma with total/peripheral anterior synechia, and cataract [4,30]. Neonatal linear skin defects were seen in four out of the six patients and varied in severity; patients 1 and 3 did not show any linear skin lesions or scars on their face and neck (Figure 4D and E). This observation is not unusual as a few reported individuals with Xp22 monosomy displayed eye abnormalities with absence of skin defects [33,34,39]. Similarly, in patients with a point mutation and small deletion in *HCCS* a variety of ocular anomalies without skin defects have been described [4,5]. To determine the phenotypic spectrum in MLS syndrome, we nowadays have to distinguish between *HCCS*- and *COX7B*-mutation positive patients. Interestingly, the four females with a *COX7B* mutation did not display microphthalmia/anophthalmia, but variable linear skin defects on the neck and face at birth [9]. Together, these data indicate that *HCCS* alterations cause variable eye and skin abnormalities, while the limited data on patients with a *COX7B* mutation suggest linear skin defects as the predominant feature.

We observed a wide range of additional clinical manifestations, including CNS anomalies such as abnormal myelination and hypoplasia/agenesis of corpus callosum and microcephaly. Cardiac defects comprise ventricular tachycardia, poor contraction of left ventricle, and histiocytoid cardiomyopathy. A few individuals had developmental delay, short stature and facial dysmorphism (Table 1). Rarely, hearing loss and anal atresia with ectopic anus and fistula, as seen in patient 5 have also been observed in other cases [13,35,40,41]. Patient 2 and one reported girl [4] carried the *HCCS* nonsense mutation c.589C > T (p.R197*). They show a remarkably similar full-blown MLS phenotype with microphthalmia, sclerocornea, linear erythrodermia, hypoplastic corpus callosum, and absence of septum pellucidum. However, patient 2 was more severely affected with anophthalmia and hypoplastic optic nerve of the left eye. She suffered from severe cardiac problems and died at age 4 months. Histiocytoid cardiomyopathy had been diagnosed, similar to a female infant with MLS syndrome who also died suddenly at 4 months of age [42].

The combination of clinical features in patient 2 clearly is at the severe end of the phenotypic spectrum in MLS syndrome. At the other end, no obvious MLS-typical sign has been observed in the three *HCCS*-mutation positive female relatives of patient 1. Although we cannot exclude the presence of mild linear skin lesions at birth in the mother, maternal aunt and grandmother of patient 1, non-penetrance in females with *HCCS* mutation or Xp22.2 monosomy has already been described [4,14]. In an Ashkenazi family, the index case and her sister showed eye anomalies and/or aplastic skin defects. The two sisters and their mother, who did not show any apparent sign of MLS carried the same small deletion in *HCCS*[4]. The same large terminal Xp deletion encompassing *HCCS* has been detected in a healthy female and her MLS-affected daughter, however, the mother was found to be a mosaic with 45,X[11]/46,X,del(X)(p22.2)[89] [14]. Thus, sex chromosome mosaicism may help to prevent the development of MLS-characteristic features in females and explain, at least in part, the high degree of inter- and intrafamiliar phenotypic variability [11,14]. In line with this, patient 3 who had unilateral ocular anomalies and no skin defects turned out to be a mosaic for a 2-bp *HCCS* deletion (c.[=/524_525delAG]). While this frameshift mutation was prominent in DNA from buccal cells, detailed analysis of leukocyte-derived DNA indicated a mixture of cells either carrying one wild-type and one mutant allele (~52%) or two wild-type alleles (~48%). Mosaicism was further confirmed by absence of the c.524_525delAG mutation in LCL-derived DNA of patient 3. For lymphoblastoid cell lines, rapid progression from polyclonality to pauciclonality or even monoclonality during cell culturing has been described [23,24]. Thus, clonal evolution likely accounts for disclosure of only *HCCS* wild-type sequence in DNA isolated from patient’s 3 *in vitro* LCL culture (Figure 3B). Indeed, about 20% of established LCLs is affected by pauciclonality/monoclonality indicating that outgrowth of a single clone of fast-growing B lymphocytes is a common phenomenon in cultured LCLs [23,24]. The apparently variable degree of mosaicism in different tissues of patient 3 might have contributed to her attenuated phenotype. Nevertheless, in a female infant with classical MLS and mosaic complex X-chromosomal rearrangements [43], mosaicism did not contribute to a mild phenotype indicating that other mechanisms account for the high clinical variability in females with *HCCS* null allele.

XCI has been discussed to contribute to phenotypic variability in MLS-affected females [17], including minor or no clinical signs. The X inactivation process starts about the time of late blastocyst or early gastrulation and inactivates one of the two X chromosomes, independent of the parental origin. The differential activity of the two X chromosomes is stably transmitted to all the descendants of a single cell and gives rise to cellular mosaicism in females [44]. Unequal inactivation of the parental alleles is known as skewing and can be the result of two different mechanisms. First, stochastic factors can cause non-random XCI in the early embryo, especially when the pool of precursor cells is limited. Secondary or acquired skewed XCI is the result of cell selection downstream of the X inactivation process [44,45]. In female carriers with Xp22 monosomy or an *HCCS* mutation, cells with an active aberrant X chromosome undergo severe respiratory problems due to disturbed mitochondrial OXPHOS as shown in yeast [4,37]. Impairment of the MRC is accompanied by a decrease in yeast chronological life span indicating that loss of *HCCS* negatively affects cell survival [37]. This finding suggests that cells with an active wild-type X chromosome reproduce faster and eventually outgrow cells expressing the *HCCS* mutation-bearing X chromosome leading to elimination of mutant cells and unbalanced/skewed XCI. Indeed, a remarkable regenerative capacity has been demonstrated in healthy cardiomyocytes of female mice with heart-specific inactivation of one *Hccs* gene copy. Hyperproliferation of healthy cardiac cells efficiently compensates for loss of 50% of *Hccs*-deficient cells at a time after XCI has been completed to ensure the formation of a functional heart [46]. Thus, proliferative advantage of cells with the normal X chromosome active that leads to increased expansion of the normal cell population may ameliorate the effects of *HCCS* deficiency and decrease the likelihood of symptoms as also seen in female carriers of severe X-linked disorders and extremely skewed XCI, such as ATR-X and Wiscott-Aldrich syndrome [47,48]. Consequently, *HCCS* mutation-positive females with no apparent MLS sign, exemplified by the three healthy female relatives of patient 1, are possibly the most favorable outcome of an effective overgrowth of cells expressing the normal X-linked allele. This demonstrates a great ability of the various tissues and organs to successfully eliminate *HCCS*-deficient dying cells during embryogenesis. In accordance with this, the cardiac phenotype in female mice with heart-specific *Hccs* deficiency primarily depends on the high proliferative capacity of the healthy cardiac cells (with the normal X chromosome active) [46].

Nevertheless, Xp22 monosomy or a heterozygous *HCCS* mutation bears the danger of developing MLS syndrome-typical features raising the question “why is this so?”. *HCCS* deficiency has been demonstrated to impair the MRC and induces overproduction of reactive oxygen species (ROS) [37]. The different ability of developing tissues and organs to cope with cells harbouring a defective OXPHOS system (as a result of an active mutant X chromosome) may account for the high clinical variability in MLS syndrome and could explain some specific clinical features, such as (histioctyoid) cardiomyopathy [49], agenesis of the corpus callosum [50], and deafness which are typically found in OXPHOS disorders [8,51]. In line with this, cardiomyopathy, ventricular dilation as well as various pathologies of the cardiac conduction system and sudden cardiac death detected in ~40% of *Hccs*-deficient female mice have been explained by the amount and localization of residual diseased tissue in the heart at birth [46]. However, microphthalmia/anophthalmia, sclerocornea, microcephaly and linear skin defects are not usually found in mitochondrial diseases [8] and are unlikely to be caused by primary OXPHOS defects. Implication of mature cytochrome *c*, the final product of the holocytochrome *c*-type synthase (HCCS) reaction, in the mitochondria-dependent cell death pathway has led to the hypothesis that the inability of *HCCS*-deficient cells to undergo cytochrome *c*-mediated apoptosis may direct cell death towards necrosis [4]. However, the recent discovery that MRC inhibition and enhanced ROS levels elicit a dramatic increase in caspase-dependent apoptosis in eyes and brain of *hccs*-deficient medaka fish may explain microphthalmia and CNS defects in MLS-affected patients [37]. The variable MLS phenotypes have been proposed to result from the different molecular responses of various tissues and organs on MRC impairment, enhanced ROS production and/or increased cell death [37]. It has been hypothesized that the particular involvement of neural crest cells explains linear skin defects restricted to the face and neck [11,25] and ocular anomalies. Indeed, extensive apoptosis leading to deficiency of neural crest cells has been shown to cause anophthalmia/microphthalmia upon influenza B virus infection during early chicken and mouse embryogenesis [52].

In summary, skewing of the XCI ratio in females with loss-of-function *HCCS* mutation represents the endpoint of a cell selection process during embryogenesis. These selection biases that occurred after primary XCI have been shown to similarly affect hematopoietic and epithelial cells suggesting that loss of some X-linked genes affects cellular growth in different cellular lineages in the same way [53]. The combination of (i) stochastic events during establishment of XCI, (ii) the genetic background, and (iii) secondary cell selection mechanisms comprising OXPHOS defects with or without enhanced cell death as the main effect has detrimental impact on specific developing organs/tissues and likely determines the phenotypic outcome in females with an *HCCS* null allele.

## Conclusions

Mutation or copy number loss of *HCCS* is associated with a wide phenotypic spectrum ranging from no clinical signs to *in utero* lethality of MLS syndrome. Somatic mosaicism could be one factor contributing to a variable phenotype, however, cell selection mechanisms including OXPHOS defect and/or enhanced cell death upon *HCCS* deficiency likely underlie the great clinical variability in this rare neurocutaneous disorder.

## Competing interests

The authors declare that they have no competing interests.

## Authors’ contributions

*HCCS* molecular and FISH analysis: SF, AJ, KK, FKK, IR, VAvR; X chromosome inactivation analysis: IR; cytogenetic analysis: SF; patient ascertainment and clinical evaluation: HLA, HF, BI, MJ, AMAL, UM, CZ; manuscript writing: KK, UM, IR, VAvR; study design: KK. All authors contributed to and approved the final version of the paper.

## Supplementary Material

Additional file 1: Figure S1 Copy number analysis of *HCCS* exons 1, 3, 4, and 6 on genomic of patient 5 and her parents. qPCR analysis revealed values that were comparable to a haploid sample (black bars) for patient 5 (orange bars) and her father (blue bars), while values of her mother (red bars) were comparable to a diploid sample (white bars). Each bar represents the mean ± SD of at least two experiments, each performed in duplicate. **Figure S2.** Delineation of the Xp22.2 deletion breakpoints in patient 1. FISH with fosmid clones out of Xp22.2 (red) and the X centromere probe *DXZ1* (green) hybridized to lymphocyte metaphase spreads of patient 1. A. G248P86973A3 showed signals (red) on both X chromosomes. B. FISH with G248P89648H11 revealed only one signal (red) on the wild-type X chromosome. C. G248P82946A7 gave signals (red) on one X chromosome. D. FISH with G248P8046H9 revealed signals on both the deleted and the wild-type X chromosome. Chromosomes were counterstained with DAPI. **Figure S3.** Copy number analysis of *HCCS* exons 2-7 on genomic of patient 1. qPCR analysis revealed values that were comparable to a haploid sample (grey bars) for patient 1 (black bars). Values of a diploid sample are indicated by white bars. Each bar represents the mean ± SD of at least three experiments, each performed in duplicate. Click here for file
