# Supplementary material for: Clinical spectrum of females with HCCS mutation: from no clinical signs to a neonatal lethal form of the microphthalmia with linear skin defects (MLS) syndrome
Source: Orphanet J Rare Dis. 2014 Apr 15;9:53. doi: 10.1186/1750-1172-9-53 (PMC4021606; doi:10.1186/1750-1172-9-53)
Supplement: Additional file 1: Figure S1 — Copy number analysis of HCCS exons 1, 3, 4, and 6 on genomic of patient 5 and her parents. qPCR analysis revealed values that were comparable to a haploid sample (black bars) for patient 5 (orange bars) and her father (blue bars), while values of her mother (red bars) were comparable to a diploid sample (white bars). Each bar represents the mean ± SD of at least two experiments, each performed in duplicate. Figure S2. Delineation of the Xp22.2 deletion breakpoints in patient 1. FISH with fosmid clones out of Xp22.2 (red) and the X centromere probe DXZ1 (green) hybridized to lymphocyte metaphase spreads of patient 1. A. G248P86973A3 showed signals (red) on both X chromosomes. B. FISH with G248P89648H11 revealed only one signal (red) on the wild-type X chromosome. C. G248P82946A7 gave signals (red) on one X chromosome. D. FISH with G248P8046H9 revealed signals on both the deleted and the wild-type X chromosome. Chromosomes were counterstained with DAPI. Figure S3. Copy number analysis of HCCS exons 2-7 on genomic of patient 1. qPCR analysis revealed values that were comparable to a haploid sample (grey bars) for patient 1 (black bars). Values of a diploid sample are indicated by white bars. Each bar represents the mean ± SD of at least three experiments, each performed in duplicate. [file 1750-1172-9-53-S1.docx]

**Supplementary Material**

**Figure S1**


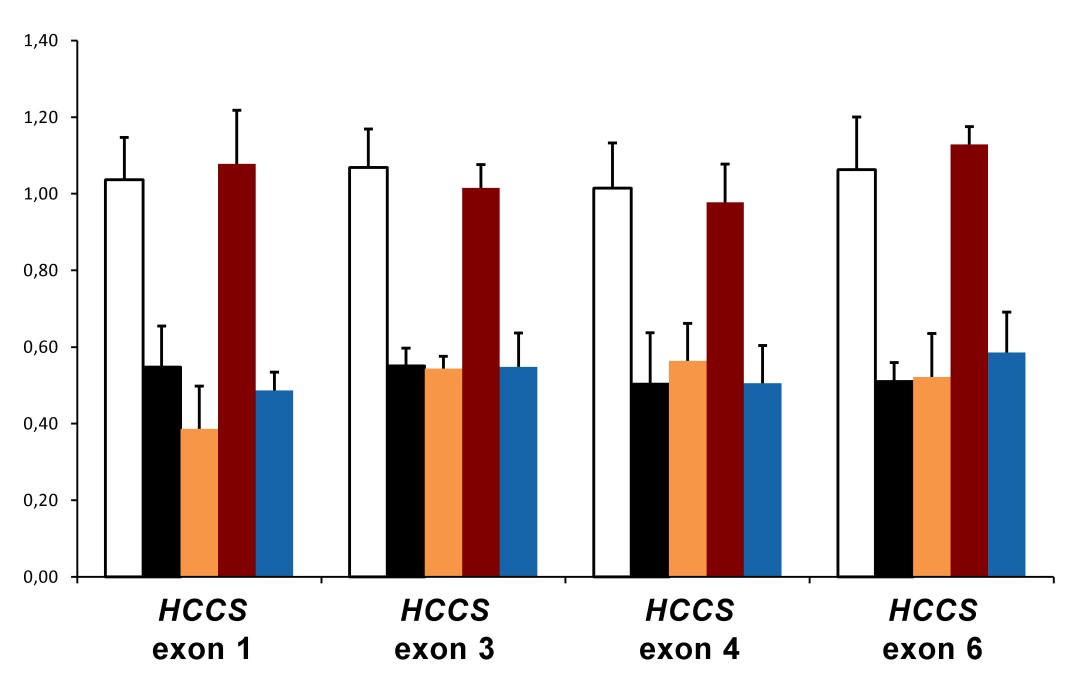


**Figure S1** Copy number analysis of *HCCS* exons 1, 3, 4, and 6 on genomic of patient 5 and her parents. qPCR analysis revealed values that were comparable to a haploid sample (black bars) for patient 5 (orange bars) and her father (blue bars), while values of her mother (red bars) were comparable to a diploid sample (white bars). Each bar represents the mean ± SD of at least two experiments, each performed in duplicate.

**Figure S2**

**A B**


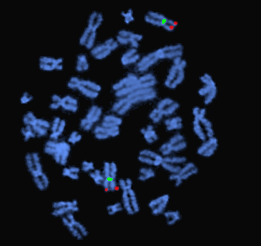

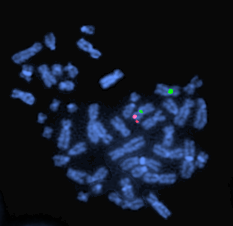


**C D**


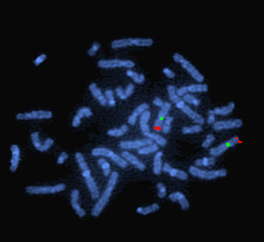

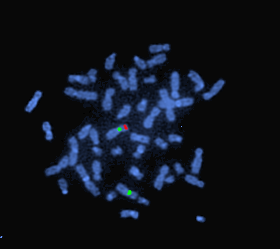


**Figure S2** Delineation of the Xp22.2 deletion breakpoints in patient 1. FISH with fosmid clones out of Xp22.2 (red) and the X centromere probe *DXZ1* (green) hybridized to lymphocyte metaphase spreads of patient 1. **A**. G248P86973A3 showed signals (red) on both X chromosomes. **B**. FISH with G248P89648H11 revealed only one signal (red) on the wild-type X chromosome. **C**. G248P82946A7 gave signals (red) on one X chromosome. **D**. FISH with G248P8046H9 revealed signals on both the deleted and the wild-type X chromosome. Chromosomes were counterstained with DAPI.

**Figure S3**


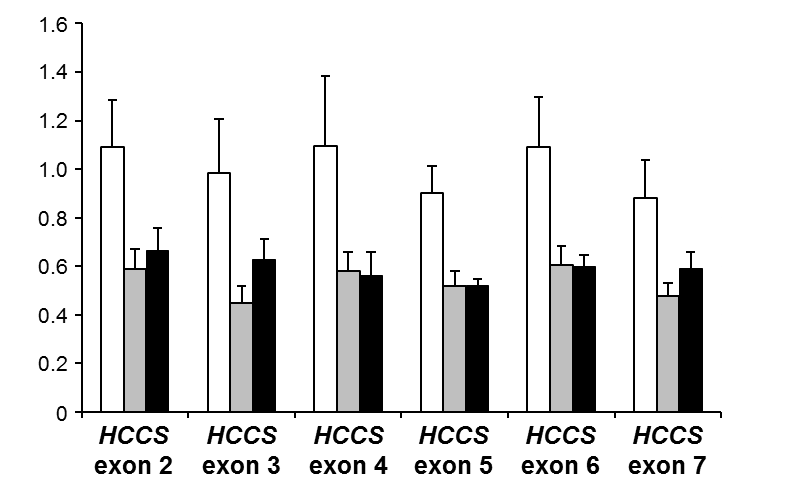


**Figure S3** Copy number analysis of *HCCS* exons 2-7 on genomic of patient 1. qPCR analysis revealed values that were comparable to a haploid sample (grey bars) for patient 1 (black bars). Values of a diploid sample are indicated by white bars. Each bar represents the mean ± SD of at least three experiments, each performed in duplicate.
